# Novel endogenous simian retroviral integrations in Vero cells: implications for quality control of a human vaccine cell substrate

**DOI:** 10.1038/s41598-017-18934-2

**Published:** 2018-01-12

**Authors:** Chisato Sakuma, Tsuyoshi Sekizuka, Makoto Kuroda, Fumio Kasai, Kyoko Saito, Masaki Ikeda, Toshiyuki Yamaji, Naoki Osada, Kentaro Hanada

**Affiliations:** 1 Department of Biochemistry & Cell Biology, National Institute of Infectious Diseases, Tokyo, 162-8640 Japan; 2 Pathogen Genomics Center, National Institute of Infectious Diseases, Tokyo, 162-8640 Japan; 3 Laboratory of Cell Cultures, National Institutes of Biomedical Innovation, Health and Nutrition, Ibaraki, Osaka, 567-0085 Japan; 4 Graduate School of Information Science and Technology, Hokkaido University, Sapporo, 060-0814 Japan

## Abstract

African green monkey (AGM)-derived Vero cells have been utilized to produce various human vaccines. The Vero cell genome harbors a variety of simian endogenous type D retrovirus (SERV) sequences. In this study, a transcriptome analysis showed that DNA hypomethylation released the epigenetic repression of SERVs in Vero cells. Moreover, comparative genomic analysis of three Vero cell sublines and an AGM reference revealed that the genomes of the sublines have ~80 SERV integrations. Among them, ~60 integrations are present within all three cell sublines and absent from the reference sequence. At least several of these integrations consist of complete SERV proviruses. These results strongly suggest that SERVs integrated in the genome of Vero cells did not retrotranspose after the establishment of the cell lineage as far as cells were maintained under standard culture and passage conditions, providing a scientific basis for controlling the quality of pharmaceutical cell substrates and their derived biologics.

## Introduction

The Vero cell lineage, a permanent cell line established from the kidney tissue of an African green monkey (AGM)^1,2^, is susceptible to various types of viruses^3^ as well as several bacterial toxins including Shiga-like toxins (or “Vero” toxins)^4^. Vero cells have pseudo-diploid karyotypes^5,6^, and are non-tumorigenic unless they are extensively passaged^7–10^. Due to these characteristics, Vero cells have been utilized in various activities against infectious diseases, serving as a biological material in research laboratories, a diagnostic tool in clinical laboratories, and also a cell substrate for human vaccines in pharmaceutical industries^11–13^. We recently elucidated the whole genome sequences of the Vero JCRB0111 (Vero 0111) subline, the available cryo-stock of which is the oldest or nearly the oldest lot (with a passage level of 115, P115, from the original primary culture started in March 1962) of Vero cells^6^. A research consortium elucidated the whole genome sequence of *Chlorocebus sabaeus* (or *Chlorocebus aethiops sabaeus*), an AGM species or subspecies^14^, from which the Vero cell lineage likely originated^6^. The genome of Vero cells was revealed to have a homozygous ~9-Mbp deletion on chromosome 12, which caused the loss of the type I interferon gene cluster and cyclin-dependent kinase inhibitor *CDKN2A* and *CDKN2B* genes in Vero cells^6^. Type I interferons are major anti-viral cytokines in the early stages of infection^15,16^, while the products of *CDKN2A*-*CDKN2B* genes act as tumor suppressors^17,18^. Hence, the 9-Mbp homozygous deletion appears to be relevant to the key characteristics of the Vero cell lineage, a continuous cell line susceptible to various virus types^6^.

In a previous study, large variations in simian type D retrovirus (SRV)-like sequences were found in the Vero cell genome^6^. SRV are known to be prevalent in many macaque monkeys in both captive and wild environments^19^. Proviral sequences homologous to exogenous SRV sequences have been identified in the genomes of a langur (*Trachypithecus obscurus*) and baboon (*Papio cynocephalus*)^20,21^. The endogenous retroviral sequences in non-human primates are generally referred to as simian endogenous retrovirus (SERV) sequences. However, the distribution of SERV sequences in Old World monkeys, including AGM, has not been thoroughly investigated.

In the life cycle of a retrovirus^22^, the viral RNA genome (commonly encodes *gag* for the group-specific antigen serving as viral structural proteins, *pol* for the enzymes including reverse transcriptase and integrase, and *env* for envelope proteins, and, in many cases, additional genes depending on virus types) is reverse transcribed to DNA, which is converted to a double-strand form and then integrated into the DNA genome of the host cell as a provirus. The provirus is transcribed into RNA from the long-terminal repeat (LTR), which serves as a multifunctional unit for transcription regulation, initiation, and termination. RNA transcripts directly or after splicing serve as mRNAs, which are translated to the precursors of viral proteins, while the full-size RNA transcript also serves as the viral progenitor genome. After the assembly of the retrovirus RNA genome with the viral proteins, the resultant complex is bound to the plasma membrane of the host cells, and bud out as a retrovirus particle. When a provirus is vertically transmitted in host animals via germline cells, this provirus is referred to as an endogenous retrovirus (ERV)^23–25^. The process of endogenization is not confined to the ancient past, and recent or ongoing endogenization has been reported^26,27^. Although mammalian genomes contain numerous copies of retrovirus-related sequences, most ERVs in the mammalian genome are inactive, functioning as neither transposable elements nor infectious agents^23–25,28^. However, ERVs may sometimes inactivate or activate nearby genes in the host cell genome, while the transcribed RNA of ERVs may directly activate the innate immune system of host cells^24,28,29^. In addition, ERVs may have cryptic potential to generate infectious virus particles after recombination or mutual complementation among different inactive proviruses^24,25,27^. Therefore, the characteristics of ERVs provide an important basis for the ensured safety of all cell-based biologics from conventional vaccines to advanced cell therapeutic agents.

In order to better understand the genomic characteristics of the Vero cell lineage from the aspect of the quality control of cells, we herein examined the whole genome sequences of two additional Vero cell sublines, Vero ATCC CCL-81 (Vero CCL-81) and Vero 76, both of which have been distributed worldwide. A comparative analysis of the genome sequences of the three Vero cell sublines and reference AGM revealed previously unknown aspects of SERV in the Vero cell lineage, which provides an insight into the biological safety of the cell line as a vaccine-producing cell substrate.

## Results

### Variations in transcribed SERV RNA in Vero cells

ERVs often have mutations that are detrimental to the retroviral life cycle, and the mutations may serve as good structural markers for ERV assignment. However, another criterion is needed for full-size retrovirus sequences inserted in the somatic cell genome in order to assess whether these sequences originated from germline-transmitted ERVs or exogenous retroviruses recently infected. A phylogenetic analysis eliminated the possibility that apparently intact SRV-like sequences found in the Vero cell genome originated from exogenous retroviruses recently infected, as described below. Thus, in the present study, we hereafter refer to SRV-like sequences found in the Vero cell genome as SERVs.

**Figure 1:**
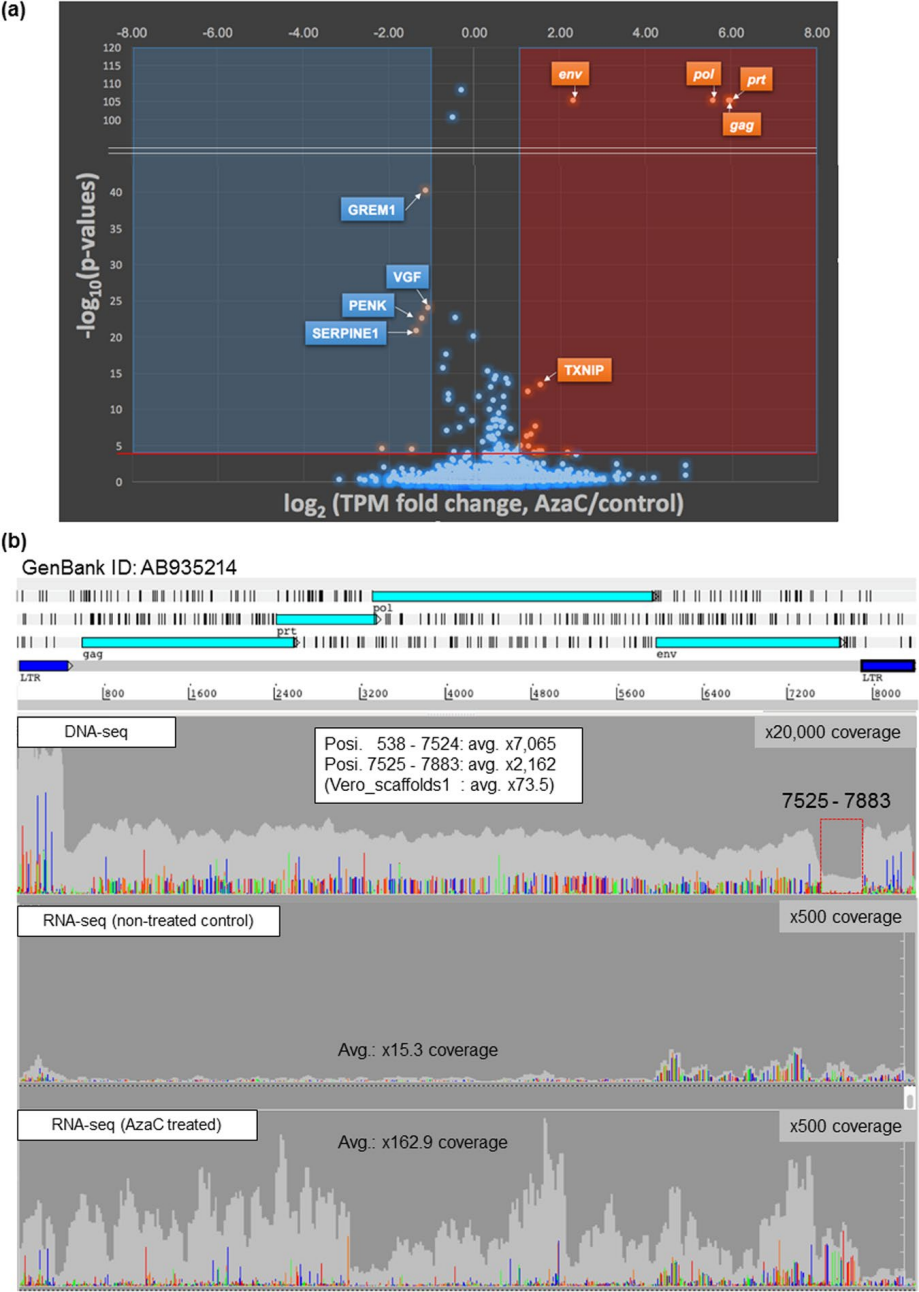
Effects of the AzaC treatment on the transcriptome of Vero cells. The mRNA-enriched fractions obtained from AzaC-treated and -untreated Vero 0111 cells were subjected to whole-transcriptome sequencing as described in the Methods. (**a**) The transcriptome analysis was performed using CLC Genomics Workbench 10.1 software. Significant open-reading frames (ORFs) were considered to be those with a False Discovery Rate (FDR)-normalized *p*-value less than 0.0001, and visualized with a volcano plot. (**b**) Vero cells were cultured with (*lower panel*) or without (*middle panel*) AzaC. SRV-related short reads from all paired-end short reads were collected with 15 complete genome sequences of SRV as reference sequences as described in the Methods. The short reads obtained were assembled, extending at a 100% identity cut-off and gap-closing between contigs. A single reasonable contig (9.2 kb) was obtained, followed by gene assignment and an LTR finding analysis, which matches an 8367-bp full-size consensus SERV sequence identified in the genome of the Vero 0111 cell subline (GenBank: AB935214). All short reads obtained were remapped to the SERV of the Vero genome sequence (which is shown in the upper part of the panels), followed by the extraction of genetic variations, as described in the Methods. As a comparison, the SERV assembly collected from the DNA-seq analysis described in our previous study^6^ is also shown (*upper panel*). Read depth is shown in light gray, while the relative levels of SNVs mismatching with the consensus SERV sequence (AB935214) are shown in the following colors (A: light green, T: red, G: orange, C: dark blue).

The previous analysis of short DNA reads showed sequence variations in SERVs in the genome of the Vero 0111 subline^6^. The transcription of ERVs is usually repressed by DNA methylation and by the repressive modifications of histones^30,31^. In order to confirm that SERVs are epigenetically silenced, we examined the effects of the DNA methyltransferase inhibitor 5-azacytidine (AzaC) on the transcriptome of Vero cells by RNA sequencing (RNA-seq) (Fig. 1a and Supplementary Table S1). The transcription of SERVs was indeed induced in response to AzaC (Fig. 1 and Supplementary Table S1), which is consistent with previous findings^32^.

In primary retrovirus transcripts, there are two common types: unspliced mRNA and singly spliced *env* mRNA^22^.The *env* gene was expressed more strongly in the absence of AzaC than other SERV genes: *gag*, *prt*, and *pol* (Fig. 1b, *middle panel*), suggesting the production of spliced *env* mRNA even in the absence of AzaC. In contrast, mRNAs encoding the latter three genes were more weakly expressed in the absence of AzaC, and were strongly induced in the presence of AzaC, resulting in more prominent AzaC-dependent mRNA induction in *gag*, *prt*, and *pol* than in *env* (Fig. 1a). Consequently, the transcriptional levels of *gag*, *prt*, *pol*, and *env* became similar after the AzaC treatment (Fig. 1b, *lower panel* and Supplementary Table S1): the mean values in TPM (transcripts per million) of *gag*, *prt*, *pol*, and *env* in the absence of AzaC were 7.3, 5.0, 7.3, and 77, respectively, while those in the presence of AzaC were 452, 319, 349, and 383, respectively (Supplementary Table S1). Most genes of the host genome were not or less responsive to AzaC than SERV genes (Fig. 1a and Supplementary Table S1), ruling out the possibility that the AzaC-dependent induction of *gag*, *prt*, and *pol* was simply due to the genome-wide activation of various promoters. As discussed below, most of the SERVs found in the Vero cell genome appear to be young ERVs. Thus, the prominent enhancement of SERV genes in response to AzaC may be due to their young age and the intact nature of their 5′ LTRs. The transcription of proviral retroviruses is generally initiated from the middle part in 5′-LTR named R-region^22,24^. However, our mapping of RNA-seq short reads showed that the expression of the entire region of the 5′-LTR of SERVs was induced in response to AzaC (Fig. 1b). Although the real reasons for this discrepancy currently remain unknown, it may be due to the involvement of upstream promoters in the transcription of the entire 5′-LTR region in response to AzaC or due to a technical limitation of resolution in the RNA-seq analysis for repeated targets. These results indicated that the AzaC treatment released the epigenetic repression of SERVs in Vero cells, presumably via DNA hypomethylation.

### Whole genome sequences of different Vero cell sublines

**Figure 2:**
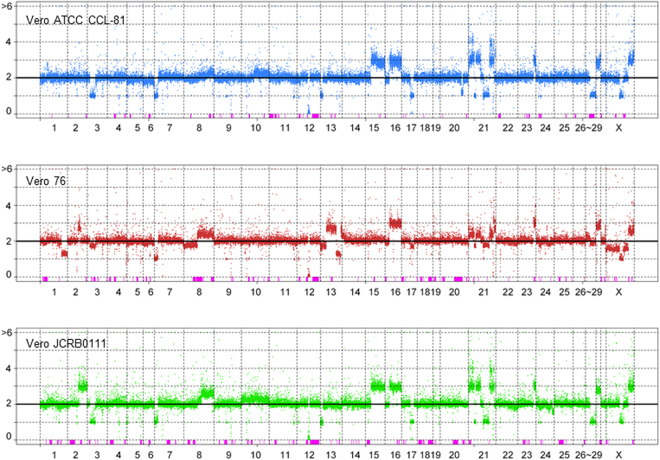
The landscape of CNV and LOH in three Vero cell lines. Each dot represents the estimated copy number in a 50 kb-length window. X- and y-axes denote chromosomal coordinates and copy numbers, respectively. The chromosome number is labeled under the panel. The boxes colored in pink represent the regions of LOH.

In order to better understand the genomic characteristics of the Vero cell lineage from the aspect of the quality control of cells, we elucidated the whole genome sequences of two additional sublines, Vero CCL-81 and Vero 76 (Fig. 2). In the Vero cell passage history, these two sublines were bifurcated at P104, while the Vero 0111 subline was bifurcated from the Vero CCL-81 and Vero 76 sublines at an earlier passage level, possibly P93 (Supplementary Fig. S1)^33–35^. Copy number variations (CNV) and loss-of-heterozygosity (LOH) were detected using FREEC software with a window size of 50 kbp. The Vero 76 subline has many heterogeneous and subline-specific copy number regions (Fig. 2). Vero CCL-81 and Vero 0111 shared more stable copy numbers than Vero 76 (Fig. 2). The large LOH on chromosome 12q, which harbors the type I interferon gene cluster, was shared among all Vero cell sublines, and this was validated by genomic PCR (Supplementary Fig. S3). In addition to the genes lost by the large deletion with LOH in chromosome 12q, we identified 185 genes that were homozygously lost in Vero cell lines (Supplementary Table S3); however, no functional bias among the 185 genes has yet been found by a Gene Ontology analysis.

A single nucleotide variant (SNV) analysis showed that the Vero76 subline has the highest number of subline-specific SNVs (Supplementary Fig. S2). The highest number of shared SNVs was observed between Vero CCL-81 and Vero 76 (Supplementary Fig. S2), which supports them originating from the same cell population. However, a relatively large number of shared SNVs were observed between any two sublines. One of the reasons for an abnormal segregating pattern may be that many new mutations have been segregated within cell populations and divergence among the different cell sublines has proceeded via a population-like process rather than a complete clonal process.

### SERV integration sites in the genome of Vero cells

A total of 499 regions homologous to SRV sequences longer than 100 bp were found in the AGM *C. sabaeus* draft genome sequence, using a consensus SERV sequence previously identified in the genome of Vero 0111 cells as a query. Among them, three regions were larger than 7000 bp and contained nearly full-length SRV-like sequences. However, all SRV-like sequences harbored at least one frameshift mutation in the protein-coding sequences.

In addition to these SRV-like sequences presented in the draft genome sequence, we identified 84 loci with the potential integrations of SRV-like sequences in the genomes of Vero cell sublines using paired-end short read information (see METHODS and Supplementary Fig. S4). The loci identified were named according to a systematic nomenclature, in which each locus was named under the prefix SVL (SERV-variation locus) followed by a chromosome number and letter (*e.g*., SVL4c corresponds to the third locus with an SERV integration in chromosome 4) as summarized in Supplementary Table S2. We identified potential sequences for target site duplication at integration sites in 78 SVLs, and also confirmed that the pre-integration sites were present in the AGM reference genome (Supplementary Fig. S4). Among 84 SVLs, 26 SVLs were also found to have SERV integrations in the reference AGM individual (individual ID: 1994-01) using the paired-end reads (Supplementary Table S2). AGM reference individual data showed the signature of target site duplication at the 26 SVLs, except for a few loci, in which additional deletions were observed (Supplementary Fig. S4). In addition, 25 out of 26 integrations in the AGM reference individual were heterozygous. These results imply that SERV integrations at SVLs were ignored during the assembly process of the draft AGM genome sequence due to their heterozygosity. Except for one locus designated as SVL27b, all of the other 83 SVLs were mapped to the same sites among all three Vero cell sublines even though the sublines have different deposition and passage histories (Supplementary Table S2). Among 58 Vero-specific SVLs, 11 SVLs were located within the intronic regions of genes (Supplementary Table S2). Fifty-seven SVLs were at least 10 kb from any protein-coding genes. The occurrence of the SVL27b integration in Vero 76, but not in Vero 0111 or Vero CCL-81was most likely due to chromosome rearrangements, not to retrotranspositions (see below). These results suggested that the retrotranspositions of SERVs in the genome of Vero cells did not occur after the establishment of the cell lineage as far as cells were maintained under standard culture and passage conditions.

In order to examine whether these Vero-specific SERV integrations were due to the specific expansion of SERV copies in the Vero cell sublines, we estimated the copy numbers of SERVs in the AGM reference individual and three Vero sublines by measuring the depth of reads mapped on the consensus SERV sequence normalized by the depth of reads mapped on the whole genome. The estimated copy numbers were 85.6, 84.9, 87.9, and 82.1 for the AGM reference, Vero 0111, Vero CCL81, and Vero 76, respectively, showing that the copy numbers of SERVs in these four genomes are similar. These results eliminated the possibility that the SERV copy number suddenly increased in the very early stages of the establishment of the Vero cell lineage.

### Variations in SERVs in Vero cells include intact SERV sequences

**Figure 3:**
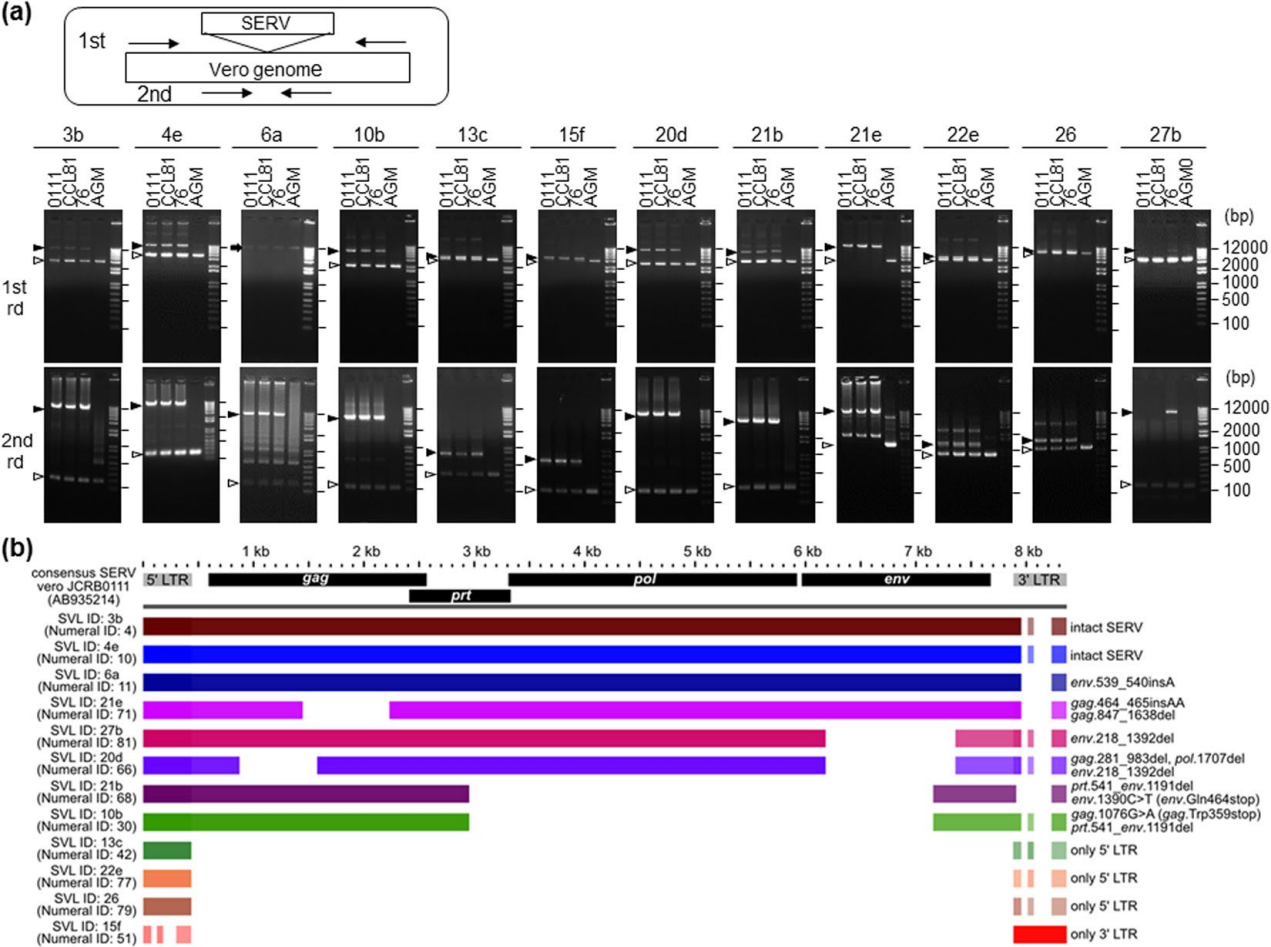
Structural comparison of SERV from different Vero sublines and different SVL. **(a)** Validation of Vero cell-specific SVL by genomic PCR. Nested PCR experiments were performed using genomic DNA from the three Vero cell sublines and AGM PBMC as described in the Methods. A schematic picture of nested PCR with the primer-binding sites is shown at the top. SVL IDs are specified under the panel. The black arrowheads indicate SERV-integrated PCR fragments, and the white arrowheads SERV-unintegrated PCR fragments. The arrow indicates an unknown band. Note that the sizes of smaller bands are consistent with the sizes expected for SERV-unintegrated genome sequences. 1st rd: 1st round PCR, 2nd rd: 2nd round PCR. All gel images shown include full-length without grouping. **(b)** A comparative analysis among twelve Vero cell-specific SVL regions. The colored bars show that the homologous region had at least 80% identity between the consensus SERV sequence found in the Vero 0111 genome (GenBank: AB935214) and each SERV region. Genetic features including mutations for respective SVLs are shown on the right side. Identity with 5′-LTR and 3′-LTR sequences of the AB935214 reference in the BLASTN homology search are: 94.3 and 73.4% for SVL13c, 72.0 and 99.0% for SVL15f, 95.4 and 74.0% for SVL22e, and 94.7 and 74.2% for SVL26, respectively. Due to the high identities with both LTRs of the reference, both 5′- and 3′-LTR regions of these SVL integrations are depicted in the panel, although the SVLs with no intervening sequence (13c, 15f, 22e, and 26) are the solo LTRs as highlighted on the right side of the panel.

In order to elucidate the SERV sequences integrated in different loci separately, each SERV was amplified by PCR for sequencing. Among 58 Vero-specific SVLs, we designed specific PCR primer sets for eleven loci, but not the other loci due to their flanking sequences being non-unique. Among the eleven SVLs, ten loci produced SERV-integrated longer fragments and SERV-unintegrated shorter fragments from the three Vero sublines, and one locus, SVL21e, only produced a long fragment (Fig. 3a). In contrast, all eleven SVLs produced only SERV-unintegrated short fragments from the DNA of AGM proximal blood monocyte cells (PBMC) (Fig. 3a). These results are consistent with our bioinformatic analysis showing that most SERV-integrations found in the Vero cell genome are Vero-specific and heterozygous (Supplementary Table S2). Sequencing of the PCR fragments with SVL integrations revealed that integrated sequences in the same SVL were identical among the three Vero cell sublines; however, the integrated sequences in different SVLs clearly varied, even in the same Vero cell subline, due to various deletions and SNVs (Fig. 3a,b). Notably, SVL3b and 4e integrations were found to encode apparently full-size retroviruses without any obvious loss-of-function mutations (Fig. 3b and Supplementary Fig. S5).

### Phylogenetic analysis of integrations at SVL3b and SVL4e

**Figure 4:**
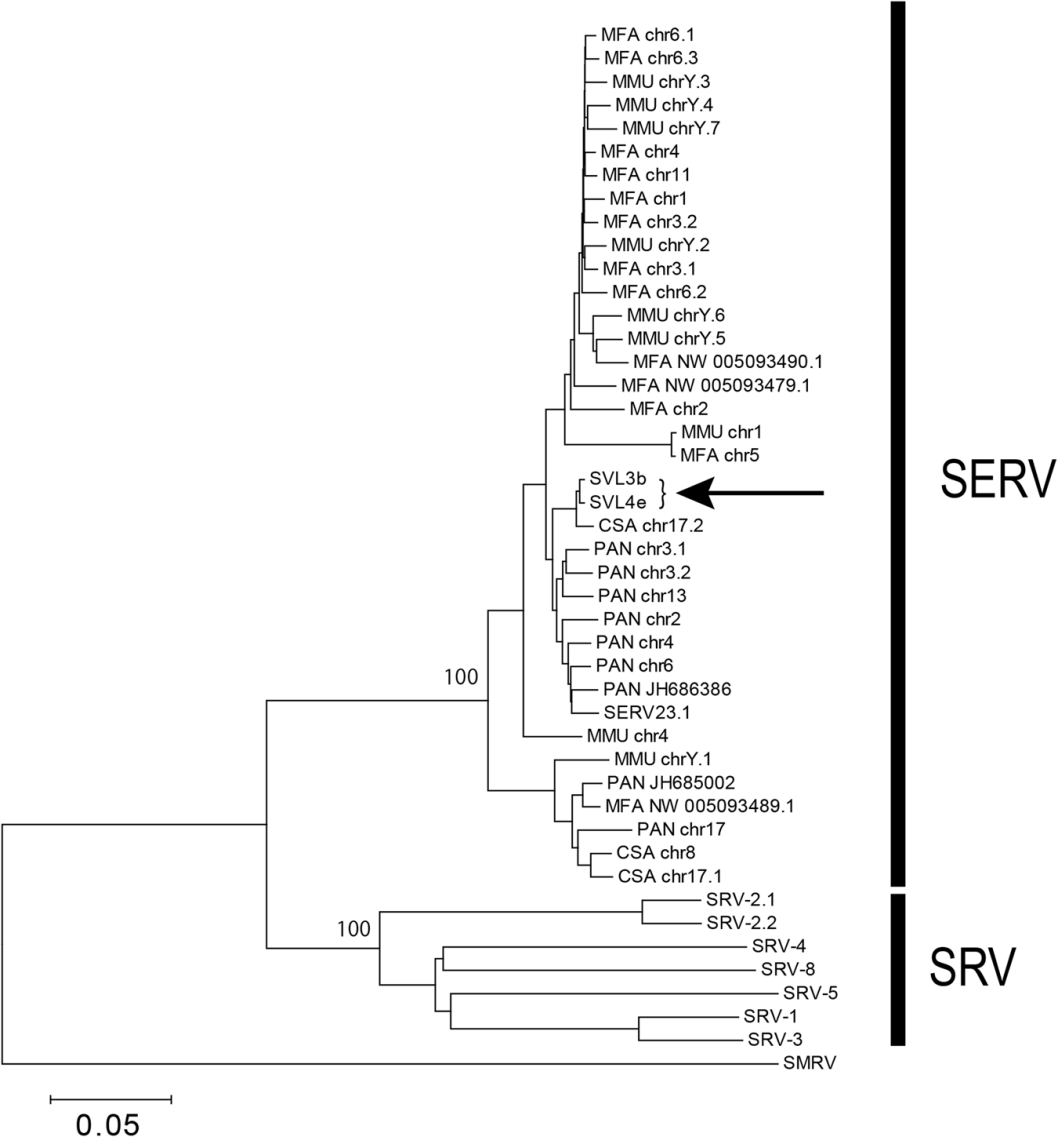
Phylogenetic tree of SRV and SERV sequences. The SRV and SERV sequences formed two distinct clades. The arrow indicates SVL3b and 4e sequences. Bootstrap values <50% were not shown in the tree. The sequence ID at each tip represents the name and chromosome/scaffold of the SERV sequence: MFA: *M*. *fascicularis*, MMU: *M*. *mulatta*, *PAN*: *P*. *anubis*, and CSA: *C*. *sabaeus*. SERV23.1 and SERV25.2 are the sequences originally reported as SERVs, identified in *P*. *cynocephalus*.

The seemingly intact feature of the integrations at SVL3b and SVL4e prompted us to examine whether they had originated from germline-transmitted SERVs or from recently infected SRVs. Therefore, we constructed a phylogenetic tree including exogenous SRV sequences previously isolated from infected animals (SRV1–5) and SERV sequences identified from the draft genome sequences of four Old World monkeys (*Macaca fascicularis*, *M*. *mulatta*, *Papio anubis*, and *C. sabaeus*), together with the integrated SERV sequences of SVL3b and 4e. The tree clearly showed that SRVs and SERVs formed distinct clades and that SVL3b and 4e integrations belong to the SERV clade (Fig. 4). These results strongly suggested that the full-size SERVs found in the Vero cell genome were really ERVs, not any remnants of contemporary exogenous SRVs. Thus, it is unlikely that 58 Vero cell-specific SVL integrations (which are absent in the AGM reference genome) were generated by infections of exogenous SRV during or after the establishment of the Vero cell lineage. The integrations at Vero cell-specific SVLs presumably existed as SERVs in the genome of the kidney cells of an AGM individual, from which the Vero cell lineage was established.

### SVL27b unique to the Vero 76 subline

Among the 84 SVLs examined in the present study, SVL27b displayed a unique complex mode of inheritance. A bioinformatic analysis indicated the existence of an integration at SVL27b in the whole genome sequences of the AGM reference and Vero 76 subline, but not in the Vero 0111 or Vero CCL-81 subline (Supplementary Table S2). Furthermore, although the SVL27b integration was only obtained from the Vero 76 subline among the three Vero cell lines tested, no SERV-containing PCR fragment for SVL27b was amplified from the control AGM PBMC used (Fig. 3a). The copy number of chromosome 27q in the Vero CCL-81 and Vero 0111 sublines was estimated to be one, while the proximal region of chromosome 27 in the Vero 76 subline showed a mosaic, corresponding to 2 or 3 copies (Fig. 2), suggesting that the heterogeneity of the SVL27b integration among Vero cell sublines and AGM individuals was generated by chromosome rearrangements in cultured cells and also by Mendelian inheritance of the locus in animals. We intend to investigate the results obtained for SVL27b in more detail in future studies.

## Discussion

The present study elucidated various characteristics of SERVs integrated into the genome of the Vero cell lineage. An RNA-seq analysis suggested that DNA hypomethylation released the epigenetic repression of SERVs in Vero cells (Fig. 1 and Supplementary Table S1). A spliced *env* mRNA of SERV was found to be expressed even in the absence of AzaC (Fig. 1). The production of ERV-derived RNA and proteins may serve as cytosolic viral alert signals that activate the innate immune system of host cells^36^. The continuous expression of spliced *env* mRNA may activate the innate immune system at a basal level, which may result in the basal tolerance of host cells to various exogenous viruses. Although type I interferons play a predominant role in the anti-viral innate immune system, the Vero cell lineage lacks the type I interferon gene cluster (Supplementary Fig. S3)^6^. The lack of type I interferon production in response to ERVs may minimize basal innate immunity, which may, in turn, play a role in the high susceptibility of Vero cells to various virus types. It is important to note that the expression of spliced *env* mRNA may generate resistance against exogenous SRVs through the pre-occupation of the cell surface receptor with the endogenous *env* protein, thereby preventing additional infections of exogenous SRVs, which share the same receptor with SERVs^37,38^.

Furthermore, comparative genomics among three Vero cell sublines and an AGM reference revealed that the genomes of the sublines have ~80 SERV integrations, but that the same integration events are shared with the three cell sublines (Supplementary Table S2). Although the initial integration of an exogenous retrovirus occurs heterozygously in the host genome, its germline transmitted sequence gradually becomes homozygous and finally becomes fixed or lost in populations by genetic drift. Therefore, old ERVs are often characterized by their homozygosity in the host cell genome^39^. Notably, most Vero cell-specific SERVs are heterozygous (Supplementary Table 2), which suggests that they are young ERVs in the evolution of Old World Monkeys, while the phylogenetic analysis showed that they had not originated from recently infected exogenous SRV (Fig. 4). In addition, the copy numbers of SERVs in the genomes of the AGM reference and three Vero sublines were similar. These results suggested that most of the Vero cell-specific SERVs had been segregated within the AGM populations and that the difference in the numbers of SERV-integrations between the AGM reference individual and Vero cell sublines was due to genetic heterogeneity among AGMs. However, further studies involving additional analyses using multiple AGM samples are needed in order to confirm whether these integrations of SERV are polymorphic in AGM populations.

This study also revealed that apparently intact retroviral integrations commonly exist in the genome of Vero cells (Fig. 3). SERVs that integrated in the genome of Vero cells may not have retrotransposed after the establishment of the cell lineage when cells were maintained under standard culture and passage conditions, as described above. However, this does not guarantee that SERVs remain silent, particularly under various stressful conditions, which may activate the transcriptional expression of SERVs (Fig. 1). Reverse transcriptase activity and SRV-like particles are produced in Vero cells exposed to the chemical inducer AzaC; however, the infectivity of SRV-like particles has not been detected^32^. It currently remains unclear why the infectivity of SRV-like particles has yet to be detected even though the genome of Vero cells harbors full-size SERV sequences. A possible explanation is that the co-expression of many defective SERVs existing in Vero cells may interfere with the maturation of infective particles, even in the presence of intact full-size SERV transcripts. Alternatively, intact SERVs in the Vero cell genome may remain silenced, even after the AzaC treatment, whereas defective SERVs are responsive. Further studies are needed in order to resolve this issue.

Deficient ERVs may serve as a natural source to generate infectious retrovirus particles after recombination or mutual complementation among different deficient proviruses or exogenously introduced DNA fragments^24,25,27^. Moreover, the present study revealed that some SERVs in the Vero cell genome have seemingly intact retrovirus sequences (Fig. 3b and Supplementary Fig. S5), suggesting their cryptic potential to act as retrotransposons or infectious agents. SERV- or SRV-derived nucleic acid fragments were detected in Vero-cell based rotavirus oral vaccines, which are currently marketed^40^, while several Vero cell-based commercialized vaccines are free of SRV-derived sequences^41^. SRV appears to be infectious to humans^42,43^. Although SRV infections in humans may be asymptomatically eliminated by a healthy immune system^42^, they may persist in immunocompromised individuals^43^. In order to guarantee the safety of Vero cell-based vaccines, manufacturers need to continually work to ensure that their products are free of SERV-derived infectious virions. This is particularly important in attenuated live vaccines, in which neither viral inactivation nor elimination may be set in their manufacturing processes. These considerations are presumably applicable to other cell substrates and their derived biologics. Thus, similar quality management against ERVs may be required for all biologics produced with any cell substrate.

## Methods

### Cell culture of Vero cells

Four Vero cell sublines, Vero 0111, Vero CCL-81, Vero 76, and Vero C1008, were used in the present study. Vero 0111 is registered with the JCRB Cell Bank as VERO under JCRB0111, which has been confirmed as free of Mycoplasma and bacteria by standard quality control in JCRB. Vero CCL-81 is registered as Vero under ATCC CCL-81. An ATCC CCL-81 cell seed culture at P125 was transferred to JCRB and registered as JCRB9013. Vero 76 is registered as VERO under ATCC CRL-1587 and JCRB9007. Vero C1008, also known as Vero 76 clone E6 or Vero E6, is registered as VERO under ATCC CRL-1586. These Vero cell sublines from ATCC have been thoroughly tested and authenticated by the ATCC. Vero cells were cultured in Eagle’s minimal essential medium supplemented with 5% or 10% fetal bovine serum at 37 °C under an atmosphere of 5% CO_2_ and 100% humidity.

### RNA-seq of SERV and other host genes

When the effects of the DNA methylation inhibitor AzaC on the transcriptome of cells are examined, cells need to be cultured in normal medium after the AzaC pretreatment in order to eliminate or minimize the side effects of AzaC on the metabolism of RNA^44,45^. Thus, the following culture protocol was used: On day 0, Vero 0111 cells were plated at 1.5 × 10^5^ cells/well (1 ml of normal culture medium per well) in a 6-well plate and cultured overnight. On day 1, AzaC (Sigma-Aldrich) was added to the cell culture at a final concentration of 313 ng/ml, but was not added to the untreated control culture. Cells were then cultured. On day 2, AzaC-untreated control cells were subjected to RNA isolation. On day 3, after medium exchange, AzaC-treated cells were cultured in AzaC-free normal medium. On day 4, AzaC-pretreated cells were subjected to RNA isolation. Total RNA was extracted and purified on RNeasy spin columns (Qiagen), according to the manufacturer’s instructions. The mRNA fraction was enriched with the FastTrack MAG micro mRNA isolation kit (Invitrogen), according to the manufacturer’s instructions. A cDNA library was then synthesized from 10 ng of enriched mRNA with the ScriptSeq v2 RNA-Seq Library Preparation Kit (Epicentre, Biotechnologies, Madison, WI) with 15 cycles of amplification according to the manufacturer’s instructions. The RNA-seq library was electrophoresed on 1% agarose gel and the library with a selected size range (250–700 bp) and specific adapters was purified with the Wizard SV Gel and PCR Clean-Up System (Promega). Two bar-coded RNA-seq libraries (control or AzaC treatment) were pooled together at an equal concentration, and the pool was subjected to 81-mer paired-end sequencing in two lanes of Genome Analyzer IIx (Illumina Inc., San Diego, CA) for whole transcriptome sequencing. Sequencing reads were obtained as follows: 0-AzaC (control): 20,104,436 pair, AzaC: 18,304,234 pair. In order to identify nucleotide variations and the redundancy of SERV, SRV-related short reads from all paired-end short reads (insert size average: 317 bp) of the Vero 0111 cell line were collected using the Burrows-Wheeler Aligner (BWA) Smith-Waterman alignment (SW) mapping technique^46,47^ with 15 complete genome sequences of SRV as reference sequences. The short reads (0.025%) obtained were assembled by platanus v1.21, followed by PRICE^48^ extending at a 100% identity cut-off and gap-closing between contigs. A single reasonable contig (9.2 kb) was obtained, followed by gene assignment and an LTR finding analysis, which suggested that the 8367-bp complete SRV genome sequence was identified. All short reads obtained were remapped to the SRV-Vero genome sequence by BWA-SW mapping, followed by the extraction of genetic variations using the Sequence Alignment/Map program (SAMtools)^47^. The transcriptome analysis was performed using CLC Genomics Workbench 10.1 software (Qiagen). All expression values were normalized by the global normalization method, and extracted to Transcripts Per Kilobase Million (TPM) with a False Discovery Rate (FDR) correction. Significant open-reading frames (ORFs) were considered to be those with an FDR-normalized *p*-value less than 0.0001, and visualized with a volcano plot^49^.

### Genome Sequencing

We obtained ~782 million and 1,125 million filter-passed paired-end reads of 125 bp from the Vero cell sublines Vero CCL-81 and Vero76, respectively, using Illumina HiSeq. 2500. These reads were mapped on the reference genome sequence of *Chlorocebus sabaeus* (NCBI accession number GCF_000409795, assembly version 1.1) using BWA mem^47^. Among these, 98.6% of Vero CCL-81 reads and 99.0% of Vero76 reads were aligned as pairs on the genome. Since the size of the genome sequence is approximately 2.8 × 10^9^ bp, average coverage was 34- and 49-fold for Vero CCL-81 and Vero76, respectively. The paired-end reads of AGM individual (1994-21) and Vero 0111 were downloaded from the public database^6,14^. All these data are paired-end reads of 100 bp. AGM data contained ~2.2 billion reads, while Vero 0111 data contained ~1.3 billion reads. The accession numbers of data are listed below.

### Identification of SERV using paired-end short reads

Genomic regions homologous to SRV sequences in the AGM *C. sabaeus* genome were searched using BLAST^50^. Segments shorter than 100 bp were filtered out. After filtering, these regions had BLAST *E* values ≤ 6.18 × 10^−10^. Segments within 50-bp intervals were merged. We defined these segments as SERV sequences in the draft AGM genome. We then searched paired-end reads in which one of the paired reads was mapped within these SERV regions and the other reads were mapped on non-SERV sequences, after filtering out mapped reads with a low mapping-quality score (MAPQ) of <40. When ≥5 reads with the same orientation were mapped within 100-bp windows, these windows were defined as a cluster of reads. If clusters with opposite orientations were detected within 500 bp, the pairs of clusters were defined as potential SERV integration regions. Since the sequencing depth was different among samples, we identified potential SERV integration sites using Vero 0111 reads and manually checked whether the signature was observed in the other samples. The zygosity of each SERV insertion site was also examined by manually checking the read alignment in the regions.

### Construction of phylogenetic trees for SERV and SRV

In addition to the AGM draft sequence genome, we searched the draft genome sequences of three other Old World monkeys (*M. fascicularis*, *M. mulatta*, and *P. anubis*) for SRV-like sequences. We identified 13, 9, and 9 SRV-like sequences larger than 7000 bp in the *M*. *fascicularis*, *M*. *mulatta*, and *P*. *anubis* genomes, respectively. These sequences are listed in Supplementary Table S5. We also included 6 exogenous SRV sequences (M11841.1, AF12647.1, M16605, M12349, FJ971077.1, and AB611707.1), two previously reported SERV sequences obtained from *Papio cynocephalus* (U85505 and U85506), and the squirrel monkey retro virus (SMRV) sequence (M23385.1) as the outgroup in the analysis^20^. Nucleotide sequence alignment and tree reconstruction were performed using MEGA7 with the Muscle alignment algorithm and the neighbor-joining method^51–53^. Bootstrap resampling was performed with 1000 iterations.

### Genomic PCR of SERVs and analysis of PCR fragments

The existence of SVL integrations in the genomes of Vero cells and AGM was verified by nested PCR experiments. The first PCR reaction was performed in a total volume of 15 μL with the following reagents: 0.5 μL of genomic DNA as the template, 3 μL of 5 × PrimeSTAR GXL Buffer (Takara), 200 μM of each deoxynucleoside triphosphate, 0.2 μM of each primer, and 0.375 U of PrimeSTAR GXL DNA Polymerase (Takara). The mixture was denatured at 98 °C for 1 min and then subjected to 30 cycles consisting of 98 °C for 10 sec, 55 °C for 15 sec, and 68 °C for 10 min. The final extension step was at 68 °C for 1 min. Reaction products were purified using the QIAquick PCR Purification Kit (Qiagen) according to the manufacturer’s instructions. A nested PCR reaction was then performed with 5 μL of 5 × PrimeSTAR GXL Buffer (Takara), 200 μM of each deoxynucleoside triphosphate, 0.2 μM of each primer, 0.625 U of PrimeSTAR GXL DNA Polymerase (Takara), and 0.5 μL of purified genomic PCR product as the template. The thermal cycle conditions of second PCR were the same as those of first PCR. PCR products were visualized after electrophoresis on 1.2% agarose gels. Specific bands were purified using the QIAquick Gel Extraction Kit (Qiagen) according to the manufacturer’s instructions. The sequences of the primers used are listed in Supplementary Table S4.

Paired-end libraries of the PCR fragments from SVL integrations (SVL ID; 3b, 4e, 6a, 10b, 20d, 21b, 21e, and 27b) were prepared for MiniSeq sequencing using the Nextera XT DNA Library Preparation Kit (Illumina) and sequenced with the MiniSeq Mid Output Kit (Illumina). The Illumina short reads of long-PCR fragments were assembled using the A5-MiSeq program^54^, followed by verification of the 5′ and 3′ end regions containing LTR regions using Sanger sequencing data. Short SERVs (<~2 kb; SVL ID; 13c, 15f, 22e, 26) were sequenced by the Sanger method. The sequences of primers are listed in Supplementary Table S4. A BLASTN homology search^50^ was performed for SERV sequences; aligned images of homologous regions were visualized with the GView server (https://server.gview.ca/)^55^ and ACT program^56^. In order to verify mutations among SERV regions, a mapping analysis against a previously deposited consensus SERV sequence found in the genome of Vero 0111 cells (GenBank: AB935214) was performed using BWA bwa-mem^46^ with the Illumina short reads of Long-PCR fragments, followed by visualization with the GenomeJack viewer software (Mitsubishi Space Software, Tokyo, Japan).

### Accession Codes

RNA-seq short reads have been deposited in the public database (accession number: DRA002256). DNA-seq short reads of whole genomes have been deposited in the SRA database with the project name of Vero Genome Project (BioProject PRJDB2865) and the following accession numbers: DRX086355 for the Vero ATCC CCL-81 subline, and DRX086356 for the Vero 76 subline. Regarding the SERV and adjacent regions reported in this study, DNA-seq short reads and assembled sequences have been deposited in the SRA database and DDBJ/EMBL/GenBank (SRA accession nos. DRX090708-DRX090729 and nucleotide sequence accession nos. LC310755-LC310788), respectively, as summarized in Supplementary Table S6.

## Electronic supplementary material

Supplementary Information

Supplementary tables
