# Supplementary material for: Novel endogenous simian retroviral integrations in Vero cells: implications for quality control of a human vaccine cell substrate
Source: Sci Rep. 2018 Jan 12;8:644. doi: 10.1038/s41598-017-18934-2 (PMC5766633; doi:10.1038/s41598-017-18934-2)
Supplement: Supplementary file 1 — Supplementary Information [file 41598_2017_18934_MOESM1_ESM.pdf]

Supplementary Information to:

Novel endogenous simian retroviral integrations in Vero cells: implications for quality control of a human vaccine cell substrate

Chisato Sakuma[1], Tsuyoshi Sekizuka[2], Makoto Kuroda[2], Fumio Kasai[3], Kyoko Saito[1], Masaki Ikeda[4], Toshiyuki Yamaji[1], Naoki Osada[4]*, and Kentaro Hanada[1*]

(CS and TS equally contributed to this study, and are the co-first authors)

* Co-correspondence to N.O. [email address: nosada@ist.hokudai.ac.jp] or K.H. [email address: hanak@nih.go.jp]

1. Department of Biochemistry & Cell Biology, National Institute of Infectious Diseases, Tokyo, 162-8640, Japan
2. Pathogen Genomics Center, National Institute of Infectious Diseases, Tokyo, 162-8640, Japan
3. Laboratory of Cell Cultures, National Institutes of Biomedical Innovation, Health and Nutrition, Ibaraki, Osaka 567-0085, Japan
4. Graduate School of Information Science and Technology, Hokkaido University, Sapporo 060-0814, Japan

Table of Contents

Supplementary materials and methods. -- 2
Legends to supplementary figures. --- 4
References associated with supplementary information -- 7
Figure S1. A history of Vero cells in Japan and the United States. -------------------------------------- 8
Figure S2. Venn diagram of SNVs found in three Vero cell sublines. ---------------------------------- 9
Figure S3. Validation of the homozygous 9-Mb deletion on 12q in four Vero cell sublines. -------- 10
Figure S4. A schematic example of a SERV integration analysis using paired-end reads. ----------- 11
Figure S5. Comparative structures of SERVs from different SVL in Vero cells ---------------------- 12

Supplementary materials and methods

Preparation of genome DNA. Genome DNA was prepared from Vero cells (at passage 126 for Vero ATCC CCL81, P30 for Vero 76, P115 for Vero JCRB0111, and P23 for Vero C1008) and PBMC of AGM using the Qiagen Blood & Cell Culture DNA kit (Qiagen GmbH, Hilden, Germany). Regarding the genome DNA of control AGM, we used our stocked sample previously prepared from the PBMC of a female AGM.

Insertion and deletion using DELLY. A deletion may be the best characteristic for the identification of different sublines among the Vero cell lineage because, unlike other types of mutations, deletions do not recover if the sequences are completely lost. Large insertions and deletions were identified using DELLY2 software[1]. RR and RV information in DELLY may be useful for screening major variants. The RR field represents the number of high-quality reads on the reference genome, while RV represents the number of high-quality reads on variant junction sites (info is written in the header in the vcf). We used the following filtering criteria: length ≥1000 bp, supporting paired-end reads (PE) ≥30, Ratio of RV to (RV+RR) ≥0.8, and genotyping quality of all samples ≥ 20. Using these criteria, no subline-specific deletions were supported by DELLY.

SNVs. SNVs were identified using VarScan software with the criteria of allele frequency >0.2 and minimum coverage >10. We also analyzed AGM 1994-0021 genome and excluded SNP sites polymorphic in the 1994-0021 genome. We identified 5,912,024 Vero-specific SNVs. In order to identify SNVs that are sublineage-specific and only shared between two sublineages (shared-by-two SNVs), we applied a statistical method with relatively stringent criteria. When variant alleles were not observed in a sample out of n reads, but were present in other samples with α variant reads and β reference reads, the probability of not observing variant alleles in n reads, $p(0|\ n, \alpha, \beta)$, was given by the following beta-binomial equation.

$$p(0|n,\alpha,\beta) = \frac{(n+\beta-1)!\,(\alpha+\beta-1)!}{(n+\alpha+\beta-1)!\,(\beta-1)!}$$

We calculated the probability for each SNV and defined subline-specific SNVs with a false discovery rate of 0.01.

Others. PCR of the 9-Mb deletion in the Vero cell genome were performed as described previously[2].

Legends to supplementary figures

Supplementary Figure S1. A history of Vero cells in Japan and the United States. Establishment of Vero cells and the lineage between Vero cell lines are schematically summarized. The Vero cell line was established by Yasumura and Kawakita (Chiba University School of Medicine in Japan) in 1962. In 1964[3], Vero cells at P93 were transferred to the National Institute of Allergy and Infectious Diseases (NIAID) in USA[4]. A seed culture at P113 was subsequently submitted from NIAID to the American Type Culture Collection (ATCC), and this was registered as Vero under ATCC CCL-81[4]. An ATCC CCL-81 cell seed culture at P125 was transferred to the Japanese Collection of Research Bioresources (JCRB) Cell Bank (previous Japanese Cancer Research Resources Bank) and registered as JCRB9013. Vero cells were also passaged in the National Institute of Radiobiological Sciences (NIRS) in Chiba, Japan from P102 to P111, and Vero cells at P111, prepared on August 21, 1985, were then deposited to JCRB in 1987 and registered as VERO under JCRB0111[5, 6]. To the best of our knowledge, it remains unclear whether the Vero cells with P102 in NIRS are a direct descendant of the seed culture with P93 which was transported to the USA. A Vero cell seed with P104 was transferred from the NIAID to the Middle America Research Unit (MARU) in Panama, its subculture pool (#76) with P124 was moved to the Communicable Disease Center (CDC) in the USA, and the subline was designated as Vero 76[4]. Several clonal derivatives from Vero 76 were separated in the CDC, and one of them, clone E6, was designated as Vero C1008[4].

Supplementary Figure S2. Venn diagram of SNVs found in three Vero cell sublines. The three circles represent the union of SNVs found in the three Vero sublines and the numbers in each colored area denote the number of SNVs classified into each category.

Supplementary Figure S3. Validation of the homozygous 9-Mb deletion on 12q in four Vero cell sublines. PCR was performed using genome DNA from the four Vero cell sublines (JCRB0111, ATCC CCL-81, Vero 76, and Vero C1008) and AGM PBMC as the template with two types of primer sets: one is the set flanking the 9-Mb deletion on chromosome 12q, and another is the set designated for the non-deleted normal genome control[2]. Note that Vero C1008 is a clonal subline derived from the Vero 76 subline (Supplementary Figure S1). Amplicons corresponding to the deletion were produced from all Vero cell sublines, but not from AGM PBMC, while amplicons corresponding to the non-deleted counterparts were produced from AGM PBMC, but not from any

Vero cell subline. Arrows indicates the primers used in genomic PCR. The gel image shown includes full-length without grouping.

Supplementary Figure S4. Detection of SERV integration and target site duplication. (A) Schematic example of SERV integration analysis using paired-end reads. The horizontal blue block represents the genome sequence and the red blocks represent SERV sequences. Paired-end sequences are indicated by the small arrows connected by the dashed lines. The SERV-integrated loci that are not represented in the draft genome sequences (orange squared regions) are identified by the cluster of reads in which one of the reads is mapped within the SERV sequences and the other reads are mapped on non-adjacent sequences to SERV. (B) Detection of target site duplication. The integration of SERV generates a 4-6-bp target site duplication (the upper panel, the green boxes). When a SERV sequence is not present in the draft genome sequence, we expect more reads to be mapped on the target site (the lower panel). (C) The target site at SVL9e. The height of grey bars in the upper panel shows the coverage of AGM reference reads and the lower panel shows the mapped reads of Vero 0111. The 6-mer target site is indicated by the green rectangle. The peak at the target site demonstrates that a SERV integration is present in the AGM reference sample, but absent in the AGM reference genome sequence. (D) The target site at SVL9b. A SERV integration is not observed in the AGM reference sample.

Supplementary Figure S5. Comparative structures of SERVs from different loci in Vero cells.

(**a**) A mapping analysis was performed with the short reads of eight SVL Long-PCR amplicons. Nucleotide substitutions were detected in all SERV regions against the consensus SERV sequence in the Vero 0111 genome (GenBank: AB935214). There is no mapping region in 3' LTR and the mapping read coverage of 5' LTR is approximately twice as high as those of other regions, suggesting that eight SERVs have strong similarities between the 5' and 3' LTR regions. The perfect identical SERV sequence was not detected among eight SVL regions. No coverage from *prt* to *env* was detected in same region of SVL21b and 10b; SVL27b and 20d also contain the no coverage

region in *env*. Read depth is shown in light gray, while the relative levels of SNVs mismatching with the consensus SERV sequence (AB935214) are shown in the following colors (A: light green, T: red, G: orange, C: dark blue). (**b**) A comparative analysis was conducted with eight SERV and the consensus SERV sequence. Two SERVs (SVL3b and 4e) and six SERVs (SVL6a, 21e, 27b, 20d, 21b and 10b) consist of complete and incomplete structures, respectively. Strong similarities were noted in 3' LTR among the eight SERVs. As described above in Fig. S4a, The SERVs containing the same deletion patterns were detected in SVL21b and 10b and in SVL27b and 20d, suggesting that truncated SERVs were duplicated in the Vero-cell chromosome.

References associated with supplementary information

1. Rausch, T. et al. DELLY: structural variant discovery by integrated paired-end and split-read analysis. *Bioinformatics* **28**, i333-i339 (2012).
2. Osada, N. et al. The genome landscape of the African green monkey kidney-derived Vero cell line. *DNA Res* **21**, 673-683 (2014).
3. Yasumura, Y. & Kawakita, Y. Studies on SV40 in tissue culture: preliminary step for cancer research in vitro in *VERO Cells: Origin, Properties and Biomedical Applications*. (eds. S. B. & T. Terasima) 1-19 (Department of Microbiology School of Medicine Chiba University, Chiba, Japan; 1988).
4. Earley, E.M. & Johnson, K.M. The lineage of the Vero, Vero 76 and its clone C1008 in the United States in *VERO Cells: Origin, Properties and Biomedical Applications*. (eds. S. B. & T. Terasima) 26-29 (Department of Microbiology School of Medicine Chiba University, Chiba, Japan; 1988).
5. Terasima, T. & Yasukawa, M. History of Vero cells in Japan in *VERO Cells: Origin, Properties and Biomedical Applications*. (eds. S. B. & T. Terasima) 22-23 (Department of Microbiology School of Medicine Chiba University, Chiba, Japan; 1988).
6. Mizusawa, H. Cell line Vero deposited to Japanese Cancer Research Resources Bank in *VERO Cells: Origin, Properties and Biomedical Applications*. (eds. S. B. & T. Terasima) 24-25 (Department of Microbiology School of Medicine Chiba University, Chiba, Japan; 1988).

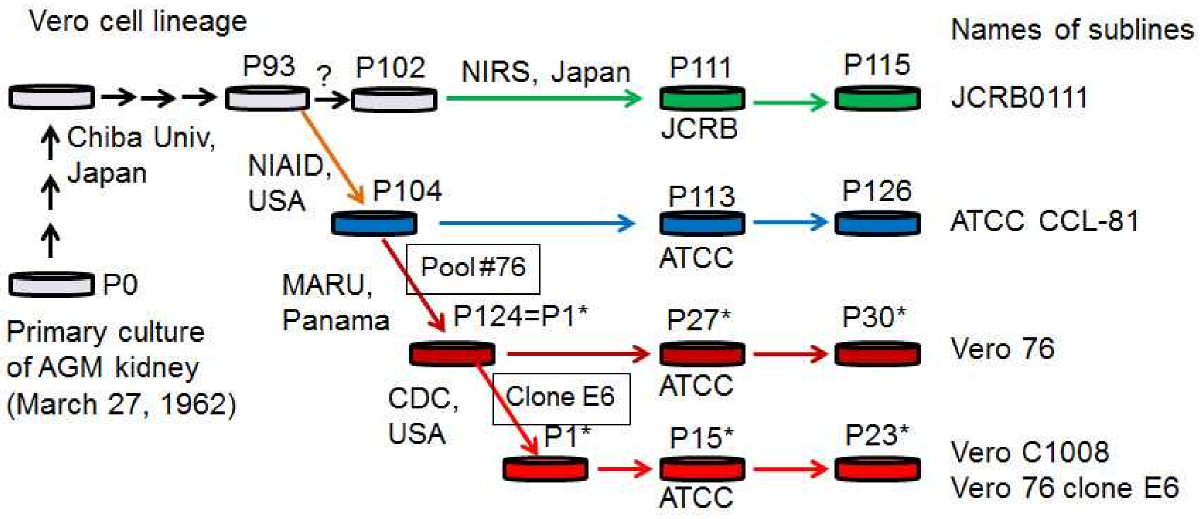
Vero cell lineage
Names of sublines
P93
?
P102
NIRS, Japan
P111
P115
JCRB0111
JCRB
Chiba Univ,
Japan
NIAID,
USA
P104
P113
P126
ATCC CCL-81
ATCC
Pool#76
P0
MARU,
Panama
P124=P1*
P27*
P30*
Primary culture
of AGM kidney
(March 27, 1962)
Vero 76
ATCC
CDC,
USA
Clone E6
P1*
P15*
P23*
Vero C1008
Vero 76 clone E6
ATCC

Figure S1

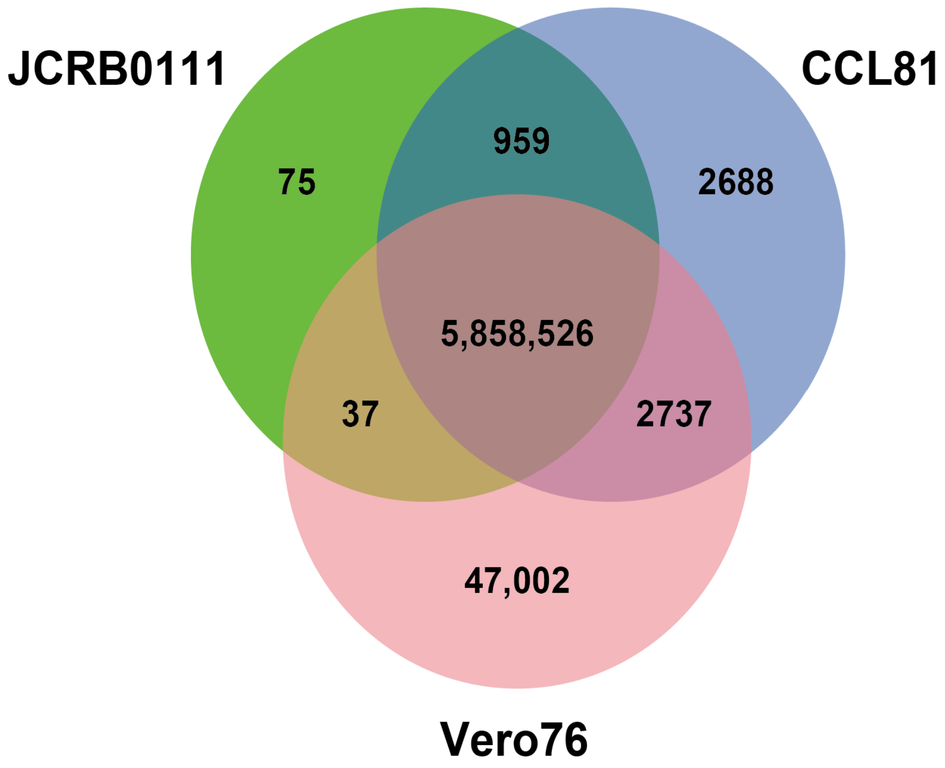

Figure S2

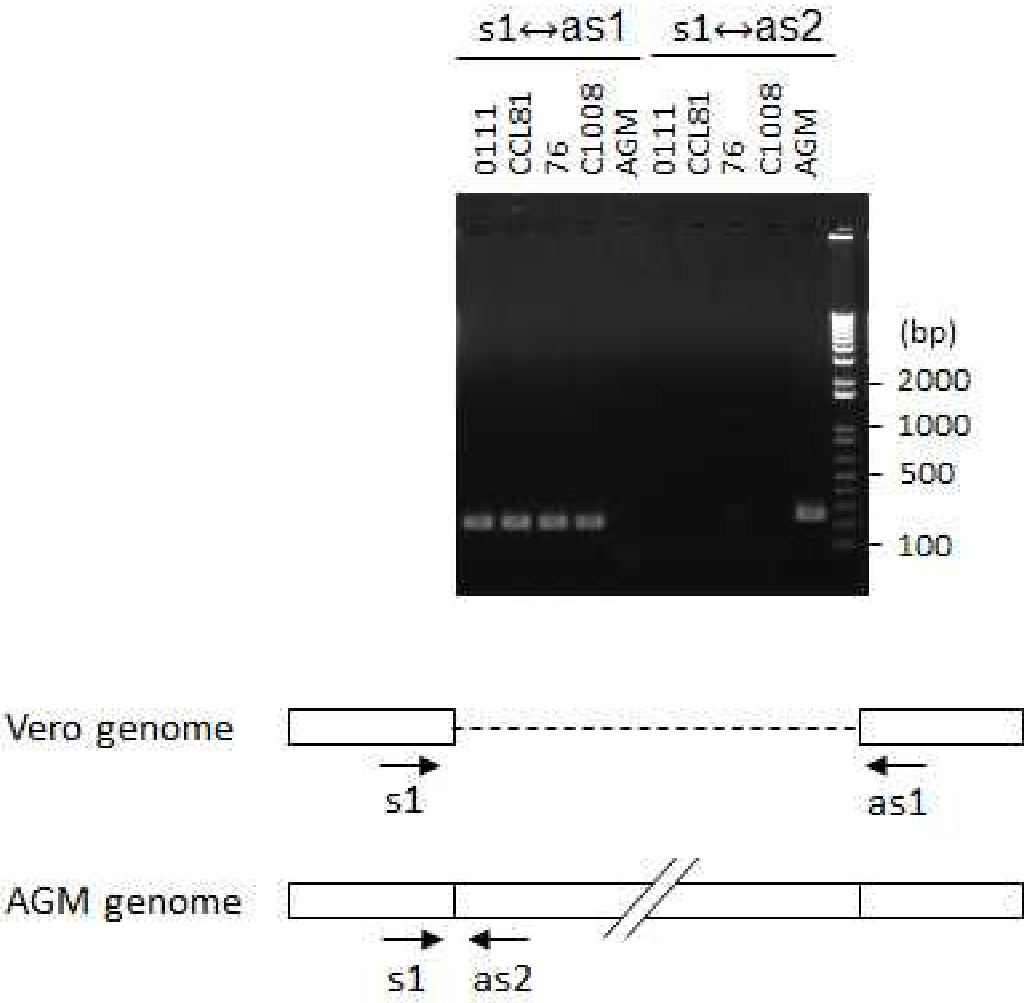
s1↔as1
s1↔as2
0111
CCL81
76
C1008
AGM
0111
CCL81
76
C1008
AGM
(bp)
2000
1000
500
100
Vero genome
s1
as1
AGM genome
s1
as2

Figure S3

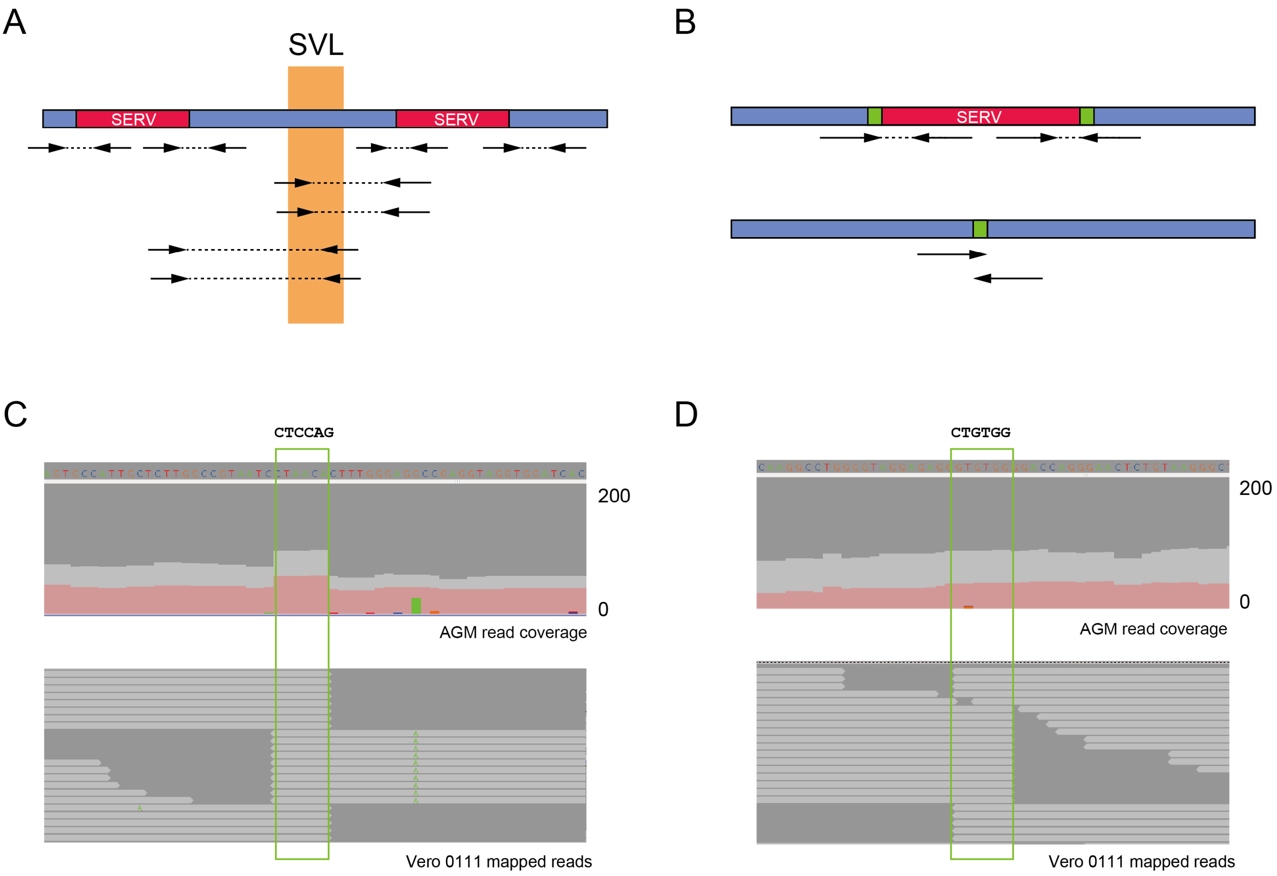
A
SVL
SERV
SERV
B
SERV
C
CTCCAG
200
0
AGM read coverage
Vero 0111 mapped reads
D
CTGTGG
200
0
AGM read coverage
Vero 0111 mapped reads

Figure S4

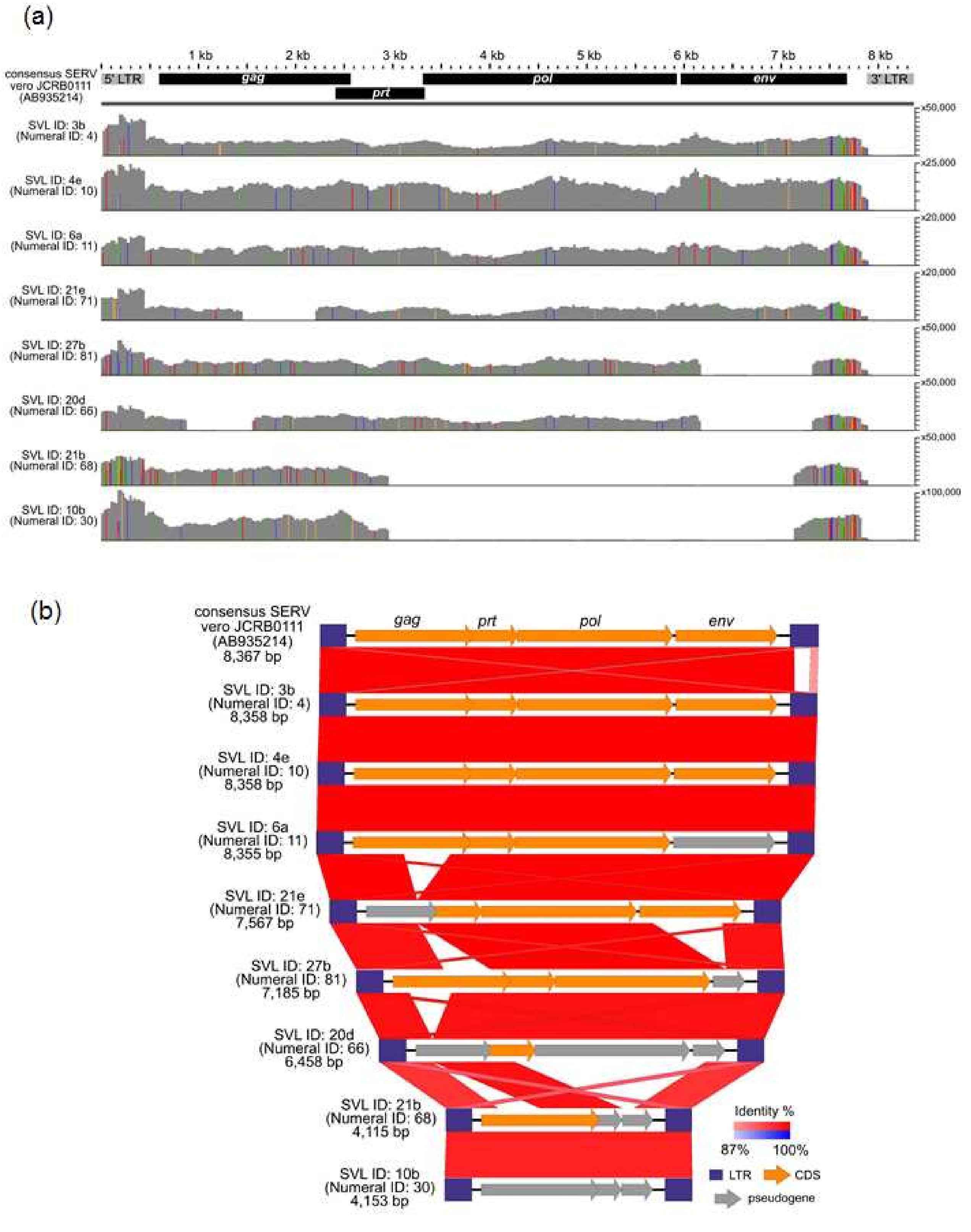
(a)
1 kb
2 kb
3 kb
4 kb
5 kb
6 kb
7 kb
8 kb
consensus SERV vero JCRB0111 (AB935214)
5' LTR
gag
prt
pol
env
3' LTR
SVL ID: 3b (Numeral ID: 4)
SVL ID: 4e (Numeral ID: 10)
SVL ID: 6a (Numeral ID: 11)
SVL ID: 21e (Numeral ID: 71)
SVL ID: 27b (Numeral ID: 81)
SVL ID: 20d (Numeral ID: 66)
SVL ID: 21b (Numeral ID: 68)
SVL ID: 10b (Numeral ID: 30)
x50,000
x25,000
x20,000
x20,000
x50,000
x50,000
x50,000
x100,000
(b)
consensus SERV vero JCRB0111 (AB935214) 8,367 bp
gag
prt
pol
env
SVL ID: 3b (Numeral ID: 4) 8,358 bp
SVL ID: 4e (Numeral ID: 10) 8,358 bp
SVL ID: 6a (Numeral ID: 11) 8,355 bp
SVL ID: 21e (Numeral ID: 71) 7,567 bp
SVL ID: 27b (Numeral ID: 81) 7,185 bp
SVL ID: 20d (Numeral ID: 66) 6,458 bp
SVL ID: 21b (Numeral ID: 68) 4,115 bp
SVL ID: 10b (Numeral ID: 30) 4,153 bp
Identity %
87%
100%
LTR
CDS
pseudogene

Figure S5
